# Thinning Effects on Biomass and Carbon Stock for Young Taiwania Plantations

**DOI:** 10.1038/s41598-018-21510-x

**Published:** 2018-02-15

**Authors:** Jiunn-Cheng Lin, Chih-Ming Chiu, Yu-Jen Lin, Wan-Yu Liu

**Affiliations:** 1 Division of Forestry Economics, Taiwan Forestry Research Institute, Taipei, 100 Taiwan; 2 Division of Forest Management, Taiwan Forestry Research Institute, Taipei, 100 Taiwan; 3 Division of Forest Utilization, Taiwan Forestry Research Institute, Taipei, 100 Taiwan; 4 Department of Forestry, National Chung Hsing University, Taichung, 402 Taiwan

## Abstract

Forests play an important role as carbon sinks by sequestrating carbon through photosynthesis. Thinning treatments have large impacts on carbon storage, in addition to strengthening quality and quantity of plantations. This study analyzed the effects of different thinning treatments on carbon stocks in both individual trees and stands of Taiwania (*Taiwania cryptomerioides*) plantations. Repeated field measurements and allometric equations were used to calculate total C storage and sequestration rates of *live* trees. The results of this study showed that the total carbon stock of stands with thinning treatments was less than that of the non-thinned stands. The non-thinned 23-year old stands had an estimated carbon stock of 96.8 Mg C ha^−1^, which is higher than the carbon stock found in either medium- (84.1 Mg C ha^−1^) or heavily-thinned (74.7 Mg C ha^−1^) treatment plots of the same age. If the objective of Taiwania plantations was to store large amounts of carbon in the young growth stage, without regard to the initial rate of storage, a better option is no-thinning. However, the medium thinned forests seem to be more promising for carbon sequestration than the no-thinned forests if a longer period is considered.

## Introduction

The rapid increase of greenhouse gases—particularly carbon dioxide (CO_2_)—is recognized as a primary contributor to global warming^1^. Therefore, reducing the CO_2_ concentration in the atmosphere is an important policy issue among governments under the Kyoto Protocol and the Paris Agreement. Since trees sequestrate carbon through photosynthesis^2^, forests play an important role as carbon sinks owing to their central position in the carbon cycle of terrestrial ecosystems^3–6^. The potential role of forests in mitigating climate change has been recognized by the Kyoto Protocol in Articles 3.3 and 3.4, in which forest carbon sequestration, mainly from afforestation and reforestation, is a significant mechanism for carbon mitigation at local, regional, national, and even global scales^1,7^.

Among forest management practices, thinning is the most commonly applied treatment for manipulating growth of plantations^8,9^. Thinning is able to decrease competition between the remaining trees and improve stand vigor, thereby effectively increasing volume growth for commercial purposes^10^. That is, it is able to promote the harvest value by increasing the marketable volume, size, and quality of the timber^11–13^. Benefits of thinning include: (1) improving forest health by removing weak, insect/disease-susceptible, and undesirable phenotypes of trees which can reduce tree mortality; (2) improving stand density can increase stand vigor and pest resistance; and (3) improving biodiversity by redistributing site components through changes in tree growth^14^. Thinning changes tree growth at both the individual tree and stand levels. Other studies also showed that thinning is able to promote growth of stands^15–17^. Thinning also causes changes of the carbon dynamics in forests through tree biomass loss and respiration. It also changes organic matter decomposition in soil because of aboveground tree removal over several years^18–21^.

This study investigated the effects of thinning treatments on carbon stock on a Taiwania (*Taiwania cryptomerioides*) plantation. The experimental design and analysis method can be easily replicated for other geneses. Taiwania is a monospecific genus and is naturally distributed in mid-elevation (500~2,600 m) mountainous areas in Taiwan. Taiwania has been widely planted over the past decades owing to its rapid growth, rare disease infections, and outstanding decay-resistance. It continuously supplies large-diameter logs with good quality for commercial building timber. Past studies have shown that thinning improves diameter growth of Taiwania, especially at the juvenile stage of the trees. Instead, in each of the first six years after trees are planted, weeding and eradicating of climbers are carried out 1–3 times, depending on tree conditions and growth. Since the seventh year, thinning and pruning have been implemented according to tree species, tree growth, and the degree of stocked stands, but their frequencies are uncertain. The effects of thinning intensities on tree growth and wood quality of this species have been widely studied^22,23^, yet there were few studies on the effects of thinning intensities on carbon stocks.

The objectives of this study were to analyze the effects of different thinning intensities—medium, heavy and no thinning—on aboveground biomass carbon stocks of a Taiwania plantation using both field measurements every two years and biomass allometric equations, and to discuss the thinning effects on the current annual carbon increment (CAI_C_) and mean annual carbon increment (MAI_C_) considering different thinning intensities. The results report effects of thinning treatment on individual tree level and stand level changes in carbon. The main contribution of this study is to calculate biomass carbon stocks using different allometric equations and carbon concentration for thinned and no-thinned plots.

## Materials and Methods

### Study site

The study site is located in Liukuei Township, Kaohsiung County, southern Taiwan (22°50′2″~23°00′3″N, 120°39′59″~120°45′2″E). The area of the study site is 2.0 ha and the elevation is approximately 1600 m. The site is managed by the Liukuei Research Center of the Taiwan Forestry Research Institute (TFRI). This site was originally a natural broad-leaved forest. After harvested in 1977, this site was replanted with Taiwania in 1979 at a planting density of 2000 trees ha^−1^. The goal of the TFRI is to cultivate Taiwania plantations to provide large-diameter logs with good quality, to improve the structure of stands and promote the quality and quantity of stands. Generally, the rotation of Taiwania was set at 80 years (Liu, 1976). The mean annual temperature during years 1986~1993 was 18.6 (16~23) °C. The mean annual precipitation during years 1986~1993 was 2280 (2150~3748) mm. The mean annual humidity during years 1986~1993 was 81 (71~86) %. About 88% of the rainfall is concentrated during the period from April to September^24^. Analysis of Variance (ANOVA) test is used to analyze if there exist statistical significant differences between different thinning plots. If the differences exist, Duncan’s multiple range test is then used to analyze the differences.

### Thinning treatment

These thinning treatments were simultaneously implemented on the study site in 1990, 11 years after planting. Before thinning treatments, the mean tree height and the diameter at breast height (DBH) of each tree in the site were measured. The basal area is estimated by measuring the average cross-section area at breast height in a unit area. It is around 40 m^2^ ha^−1^ before thinning. The thinning intensities were determined by post-thinning stand density. The thinning densities for medium thinning and heavy thinning are around 20% and 30%, respectively. The study plantation was thinned with 3 intensities (no thinning, medium thinning, and heavy thinning) at the tree age of 11 years, and the investigation of growth variables was conducted at the ages of 11, 13, 15, 17, and 23 years. The three treatments of thinning intensities were individually replicated 12 times. Therefore, the study plantation was divided into 36 smaller plots, each being 0.04 ha (20 × 20 m). The remaining area is the buffer zone with area of 0.56 ha.

### Estimation of biomass and carbon stock

In order to avoid too many sample trees being cut down, the most common method to estimate stand biomass is using regression models. The natural logarithmic equation shown in Equation (1) is the most commonly used allometric equation for the relationship between biomass and DBH^25–27^:

1 $$\mathrm{ln}(W)=a+b\,\mathrm{ln}\,(DBH)$$

where *W* is biomass (kg), including various biomass components of leaves and twigs, branches, dead branches, and the bole; *a* and *b* are coefficients; and *DBH* is the diameter at breast height (cm). The regression coefficients of the allometric equations for various biomass components of trees in the no-thinning and thinned stands are shown in Lin *et al*.^28^. This study measured the DBH of each tree in 36 study areas, and then adopted the regression coefficients in Lin *et al*.^28^ to obtain the biomass amount of each tree.

In this study, the aboveground stand biomass of the Taiwania plantation was calculated by determining the individual tree biomass and then multiplying this value by the stand density. The individual trees biomass of Taiwania was estimated from the DBH using an allometric regression equation in Equation (1), which contains each aboveground component of the tree and was developed by Lin *et al*.^26,28^ for the Taiwania plantation from sample trees in the neighborhood of the study site of this study (in fact, in the same range of 12 forest compartments of Liukuei Research Center of the TFRI).

Using the same principle, the stand carbon stock was also obtained by using the carbon stock of an individual tree multiplied by the stand density. The carbon stock amount of an individual tree in each study plot was calculated by the biomass values of different components multiplied by the carbon concentration of each corresponding component. 24 samples were collected in 2003. They were analyzed through an elementary analysis system (Elementar Analysen-syeteme, Hamburg, Germany) to measure the carbon concentration (%) of bole on growth cone axon samples of trees with different ages and treatments.

Through the ANOVA analysis, carbon concentrations of different thinning treatments did not differ significantly, and the average was 47.51%, which is the same with the result for carbon concentration of bole in Lin *et al*.^26^. Because Lin *et al*.^26^ included the carbon concentrations of other components such as leaves and twings, branches and dead branches, this study is directly referred to the result conducted by Lin *et al*.^26^. The carbon concentration of each corresponding components of Taiwania was measured by Lin *et al*.^26^.

Therefore, we used the following equation to calculate stand carbon stock at a specific stand age:

2 $$S{C}_{t}={N}_{t}\times \sum _{i}({W}_{i}\times {C}_{i})$$

where *SC*_*t*_ is the total amount of stand carbon stock (Mg C ha^−1^) at age *t*, *N* is stand density (trees ha^−1^) at age *t*, *W*_*i*_ is the biomass values of different components (leaves and twigs, branches, dead branches, and boles) for tree *i*, and *C*_*i*_ is the carbon concentration (%) of each corresponding component for tree *i*. To keep the contents concise, we only focus on carbon and exclude the presentation of biomass in the following discussions. Biomass can be calculated by multiplying estimates of carbon by a conversion factor of 2.1048.

### Estimation of the mean annual increment of carbon

This study used the current annual increment and the mean annual increment to estimate the gross accumulation trends for carbon in stands. The current annual increment for carbon (CAI_C_) is the increment of carbon over a period of one year at a specific age. The function can be expressed using CAI_C_ = Carbon (*t*) − Carbon (*t* − 1). The mean annual increment for carbon (MAIc) is the increments of carbon over the whole period from planting to a specific age. Expressed as a function, MAI_C_ = Carbon (*t*)/*t*. The MAI_C_ was calculated annually from the ages of 12 to 23, initiated after the thinning treatments which began at the age of 11.

## Results

### Growth variables of treatments with different thinning intensities

**Figure 1:**
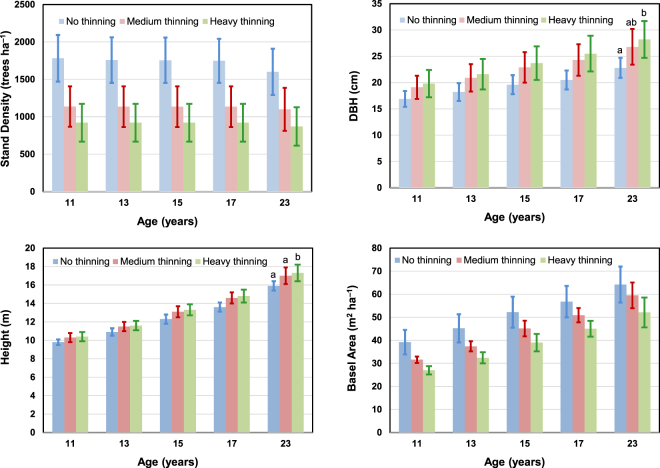
Stand characteristics of three plots after different thinning treatments between ages 11 to 23. Difference letters over bars are significantly different by Duncan’s multiple range test (p = 0.05). The statistically tests are for the pre-treatment values.

As shown in Table 1, the mean stand densities of each stand before thinning (at the age of 11 years) were 1782 trees ha^−1^ for the stand that was not be thinned, 1689 trees ha^−1^ for the stand to be medium thinned, and 1750 trees ha^−1^ for the stand to be heavily thinned. Medium and heavy thinning regimes resulted in removals of 552 and 829 trees ha^−1^, respectively. The average stand density across all three stands was 1748 trees ha^−1^. Before thinning, the average stand density was 1748 tree ha^−1^; the average DBH was 17.1 cm; and the average height was 9.9 m. After thinning, the stand density, DBH, and height for the medium and heavily thinned stands were 1137 and 921 trees ha^−1^, 19.1 and 19.8 cm, 10.3 and 10.4 m, respectively. It is shown that the three treatments have no significant differences in stand density, DBH, and height before thinning, according to the *F*-test and *p*-value (in Table 1) using Duncan’s multiple range test. This indicated that the stands were in similar condition prior to the treatments and any changes after thinning probably resulted from the treatments. The stand densities for each stand after thinning in the same year decreased to 1137 and 921 trees ha^−1^ for medium and heavy thinning, respectively (Fig. 1). The stand densities at the age of 23 years (i.e., over 12 years after thinning at the age of 11 years) were 1601, 1099, and 871 trees ha^−1^ for the non-, medium-, and heavy-thinning treatments with average mortalities of 10.2%, 3.3%, and 5.4%, respectively. The results showed that the stand with medium- and heavy-thinning treatments had lower mortality than the non-thinning treatment.

**Table 1:** Plot characteristics after different thinning treatments. Note that the statistically tests are for the pre-treatment values.

Treatments	Stand density (trees ha^−1^)	DBH^1)^y (cm)	Heighty (m)	Basal area^3)^y (m^2^ ha^−1^)	Volume^4)^y (m^3^ ha^−1^)
All plots					
Before thinning	1748 ± 280	17.1 ± 1.7	9.9 ± 0.4	39.7 ± 5.7	176.6 ± 29.4
No thinning plots	1782 ± 311^2)^	16.9 ± 1.6	9.8 ± 0.3	39.3 ± 5.3	173.7 ± 26.8
Medium thinning plots					
Before thinning	1689 ± 259	17.4 ± 1.7	9.9 ± 0.4	39.7 ± 6.3	177.8 ± 31.6
After thinning	1137 ± 270	19.1 ± 2.2	10.3 ± 0.5	31.6 ± 1.4	146.8 ± 11.3
Heavy thinning plots					
Before thinning	1750 ± 286	17.1 ± 1.9	9.9 ± 0.4	39.9 ± 6.0	177.8 ± 32.3
After thinning	921 ± 252	19.8 ± 2.6	10.4 ± 0.5	27.0 ± 1.8	127.1 ± 13.0
Duncan’s multiple range test					
*F*-test	0.492	0.381	0.342	0.015	0.446
*P*-value	0.616	0.686	0.713	0.985	0.642

^1)^DBH, diameter at breast height.

^2)^Mean ± SD (standard deviation).

^3)^Basal area = (DBH/200)^2^ × π × Stand density.

^4)^Volume = Basal area × Height × Volume form factor (0.45).

The mean DBH during the 12 year study consistently increased from 16.9 to 22.8 cm, 19.1 to 26.8 cm, and 19.8 to 28.2 cm for non-, medium-, and heavy-thinning treatments with average annual increments of 0.5, 0.6, and 0.7 cm, respectively. The stand with heavy-thinning treatment had a larger DBH increment than the other treatments. The total increasing rate of mean DBH during the 12 years for non-, medium-, and heavy-thinning treatments were 36.3, 39.4, and 42.3%, respectively. Using the Duncan multiple range test, the mean DBH differed significantly between heavy-thinning and non-thinning regimes. However, the medium-thinning showed no significant difference from non- and heavy-thinning.

The height growth in the study period did not significantly differ between treatments. The mean tree heights at the age of 23 years were 15.9, 17.0, and 17.3 m for the non-, medium-, and heavy-thinning treatments with total increments of 6.1, 6.7, and 6.9 m, respectively. The total increasing rate of height growth during the 12 years for non-, medium-, and heavy-thinning treatments were 62.3, 63.9, and 66.2%, respectively. Using the Duncan multiple range test, the height growths were significantly different between the three thinning treatments, in which heavy-thinning showed greatest growth compared to the others. The annual basal area increment rates of stands were 2.1, 2.3, and 2.1 m^2^ ha^−1^ for the non-, medium-, and heavy-thinning treatments, respectively.

### Estimation of the aboveground carbon stock of individual trees and stands

**Figure 2:**
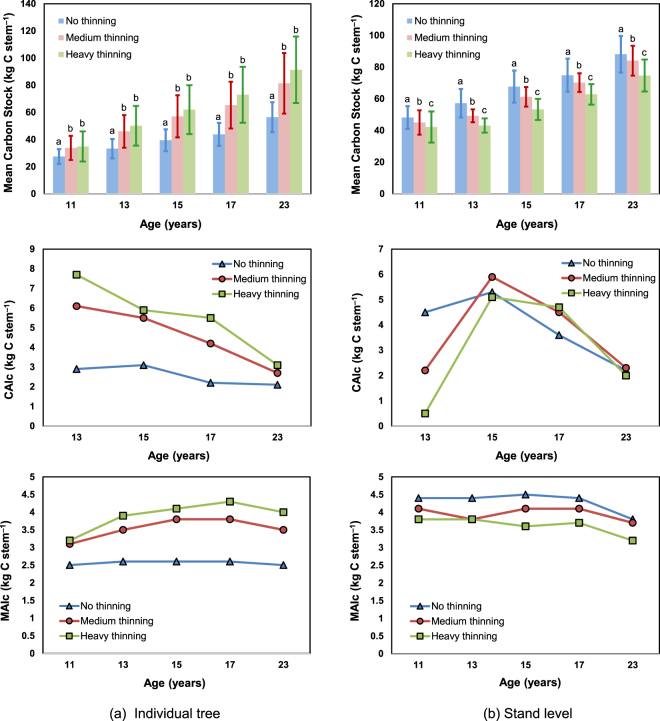
Aboveground carbon stock of three thinning treatments after thinning for (**a**) individual trees and (**b**) stands. Note that CAI_C_ denotes current annual carbon stock increment, and MAI_C_ denotes mean annual carbon stock increment. Difference letters over bars are significantly different by Duncan’s multiple range test (p = 0.05).

Figure 2 shows that the mean accumulated carbon stocks of individual trees from the ages of 11 to 23 years were 29.0 (increasing from 27.5 to 56.5), 47.6 (increasing from 33.8 to 81.4), and 56.5 (increasing from 34.9 to 91.4) kg C stem^−1^ for the non-, medium-, and heavy-thinning treatments, respectively. These differences are significant with p values less than 0.01. The largest carbon stock of an individual tree was estimated to be 91.4 kg C stem^−1^, which was in the heavily-thinned treatment plots at the age of 23 years. Note that only the aboveground component of the tree is considered for estimating the carbon stock because of the limitation of the measurement. Therefore, the carbon stock at the age of 11 was to evaluate the amount for individual trees, but the carbon stock for thinning was evaluated for only boles.

Using the Duncan multiple range test results, in individual trees, the mean carbon stock showed some difference across thinning regimes: heavy thinning showed higher mean carbon stocks than the non-thinned individual trees between the ages of 11 to 13; the medium- and heavily-thinned trees also showed higher mean carbon stocks than the non-thinned trees at the ages of 15 to 23. Carbon stock accumulation trends were also analyzed by using the CAI_C_ and the MAI_C_. In individual trees, the results showed that the CAI_C_ in the non-thinned plots had lower values for each compared year than the plots with medium- and heavy-thinning treatments. The maxima of CAI_C_ for all three treatments appeared at the age of 13 years. The highest value of CAI_C_ in this study was 7.7 kg C stem^−1^ at the age of 13 years with heavy-thinning. The maximum MAI_C_ in non-thinned individual trees appeared at the ages of 13 to 17. The maximum MAI_C_ of the individual trees with medium-thinning appeared at the ages of 15 and 17, and both results were lower than the maximum of 4.3 kg C stem^−1^ with heavy-thinning at the age of 17.

The aboveground accumulated carbon stock of the stands from the ages of 11 to 23 were 39.9 (increasing from 48.2 to 88.1), 39.1 (increasing from 45.0 to 84.1), and 32.5 (increasing from 42.2 to 74.7) Mg C ha^−1^ for the non-, medium-, and heavy-thinning treatments, with mean annual increment of 3.8, 3.7, and 3.2 Mg C ha^−1^, respectively. The largest stand carbon stock was estimated to be 88.1 Mg C ha^−1^, which was in the non-thinned treatment plots at the age of 23 years. The stand carbon stock at the same age in the medium- and heavily-thinned treatment plots were 84.1 and 74.7 Mg C ha^−1^, which were 95.5 and 84.8% of the non-thinned treatment plots, respectively. These differences are significant with *p* values less than 0.01.

Using the Duncan multiple range test results, in stands, the mean carbon stock consistently showed significantly different results among the three treatments, with non-thinned stands showing the greatest mean carbon stock, then medium-thinned stands, and then heavily-thinned stands between the ages of 11 and 23. In stands, the results showed that the CAI_C_ in the non-thinned plots had higher values at the age of 13 compared to plots undergoing medium- and heavy-thinning. The maxima CAI_C_ of the medium- and heavily-thinned plots appeared at the age of 15; the highest CAI_C_ result was 5.9 Mg C ha^−1^ with medium-thinning at the age of 15. The stand MAI_C_ of the non-thinned plots had higher values than plots with medium- and heavy-thinning for each compared year. The maximum MAI_C_ for non-, medium-, and heavy-thinning were 4.5, 4.1, and 3.7 Mg C ha^−1^, respectively, and appeared at the ages of 15, 15, and 17, respectively.

## Discussion

Although the remaining trees on the thinned plots had more rapid-growth effects—especially for individual trees—than the trees on the non-thinned plots, ultimately the total carbon stock of stands with thinning treatment proved less than that of the non-thinned stands. The effect of thinning was indeed much stronger on individual trees than that at the stand level. It is because the studied trees are still young and thinning facilitates growing of the individual trees. Thinning promotes growth of the remaining individual trees by reducing competition and increasing light and nutrient availability^29^. In contrast, thinning still results in some reduction of carbon stock so it takes a period of time to recover. From the CAI_C_ and MAI_C_ analyses, we find that the carbon stock accumulation in stands with medium- and heavy-thinning (the intersection point of CAI_C_ and MAI_C_) was shifted to the later years compared with the non-thinned stands. At what point the stands could balance or exceed the sequestrated carbon lost after thinning remains uncertain and the long-term effects of thinning on carbon stock would require additional years of measurements to determine.

In this study, thinning did not increase net carbon stock at the stand level at young ages. Other research articles had similar results. Schroeder^30^ for thinning treatment of loblolly pine (*Pinus taeda*) plantations found that thinning did not increase net carbon stocks and Schaedel^31^ found that early forest thinning only changed aboveground carbon distribution among pools. Alvarez *et al*.^31^ showed that thinning has a negligible effect of thinning intensity for Scots pine (*Pinus sylvestris L*.) and Pyrenean oak (*Quercus pyrenaica Willd*.). Dewar and Cannell^32^ simulated thinned plantations of *Pinus sitchensis* which stored about 15% less carbon than non-thinned plantations. Row^33^ also found that after thinning, the total carbon stock was lower in a 50-year loblolly pine rotation than that in non-thinned stands. Strich^34^ obtained an insignificant result on the total growth and carbon stock impacted by different thinning practices on forest growth studies. Vesterdal *et al*.^35^ investigated the carbon accumulation in Norway spruce (*Picea abies*) stands, and found a negative linear correlation with thinning intensity. He found significant decreases in aboveground tree carbon storage of 27% and 22% owing to heavy- and medium-thinning compared with non-thinned stands^36^. Li *et al*.^37^ also discovered that the total carbon stock declined with thinning for larch forests; and Ruiz-Peinado *et al*.^38^ showed that non-thinned stands had the highest carbon stocks for Scots pine (*Pinus sylvestris L*.).

In contrast, some studies indicated that thinning can increase carbon storage. Schroeder^30^ showed that thinning increased carbon stocks by 11% over 50 years for densely packed Douglas fir (*Pseudotsuga menziesii*) plantations. Balboa-Murias *et al*.^39^ showed that prolonging the rotation, selecting better quality sites, and reducing the thinning intensity increased carbon pools of radiate pine (*Pinus radiata D. Don*). It is possible that the 12-year study period of this Taiwania experiment was not long enough, although even in the short term, the more-vigorous growth of the remaining living trees in both thinning treatment stands could still not compensate for the lost carbon stock, which were 18.6% and 29.8% of the initial pre-thinning carbon stock, respectively. Generally, the rotation of Taiwania was set at 80 years^40^, and these Taiwania plantations had obviously not yet reached a mature stage.

From the last column for volume in Table 1, the average amounts of harvest^13^ for medium- and heavy-thinning treatments are 31.0 (=177.8 – 146.8) and 50.7 (=177.8 – 127.1) m^3^ ha^−1^, respectively. Through the coefficients of the equation for characterizing the relation between DBH and aboveground biomass in Lin *et al*.^28^ and the carbon concentration of boles in Lin *et al*.^28^, the C conversion factor (per air dry density) (Mg C/m^−3^) is calculated to be 0.25. With the above information, the carbon stocks of the thinned timber with medium- and heavy-thinning treatments are 7.8 (=31.0 × 0.25) and 12.7 (=50.7 × 0.25) Mg C ha^−1^, respectively.

Summary of this study is stated as follows. The objectives of this study were to analyze the effects of different thinning treatments—medium-, heavy- and non-thinning—on aboveground carbon stocks of a Taiwania plantation using both field investigative data and biomass allometric equations. Note that the field measurements are referred to a quite short period. The results of this study showed that thinning increased carbon stocks of individual trees, but did not increase carbon stocks at stand level in a Taiwania plantation. The mean accumulated carbon stock of individual trees from the ages of 11 to 23 years were 29.0, 47.6, and 56.5 kg C stem^−1^ for the non-, medium-, and heavily-thinned plots, respectively. The carbon stock of stands at the same age in the non- medium- and heavily-thinned treatment plots were 84.1 and 74.7 Mg C ha^−1^, which were 96.5 and 84.8% of the non-thinned treatment plots, respectively.

If the objective of Taiwania plantations is to store large amounts of carbon in the young growth stage, without regard to the initial rate of storage, then a better option is no-thinning. Considering the increase of carbon stocks with medium- and non- thinning treatments may not be remarkable, the medium thinned forests seem to be more promising for carbon sequestration than the no-thinned forests. The medium thinned forest had a larger carbon stock increment and is very likely to surpass the no thinned plot in a few years by observing that the peak of growth had shifted to a later years in thinned plot.

## References

[1] Houghton, J. T. *et al*. (eds) Climate change 2007: *The scientific basis* (Cambridge University Press, 2007).

[2] Watson, R.T. *et al*. (eds) *Land use, land-use change, and forestry* (Cambridge University Press, 2000).

[3] Sedjo RA (1990). The global carbon cycle: are forests the missing link?. J. Forestry.

[4] Englin J, Callaway J (1993). Global climate change and optimal forest management. Nat. Resour. Model..

[5] Schulze ED (2003). Climate change - making deforestation pay under the Kyoto Protocol?. Science.

[6] Liang F, Jia Z, Ma L (2013). The effects of thinning on carbon stocks and fluxes in a Chinese arborvitae plantation. The Forestry Chronicle.

[7] Kim C, Son Y, Lee WK, Jeong J, Noh NJ (2009). Influences of forest tending works on carbon distribution and cycling in a Pinus densiflora S. et Z. stand in Korea. For. Ecol. Mgmt..

[8] Li RS (2017). Thinning effect on photosynthesis depends on needle ages in a Chinese fir (Cunninghamia lanceolata) plantation. Sci. Total. Environ..

[9] Schaedel MS (2017). Early forest thinning changes aboveground carbon distribution among pools, but not total amount. For. Ecol. Mgmt..

[10] Liu WY, Lin CC, Su KH (2017). Modelling the spatial forest-thinning planning problem considering carbon sequestration and emissions. For. Policy. Econ..

[11] Wargo, P. A. & Harrington, T. C. Host stress and susceptibility in *Armillaria root disease, agriculture handbook No. 691* (eds Shaw, C. G. & Kile, G. A.) 88–101 (US Department of Agriculture, 1991).

[12] Gottschalk, K. W. Using silviculture to improve health in northeastern conifer and eastern hardwood forests in *Forest health through silviculture* (Ed. Eskew, L. G.) 219–226 (US Department of Agriculture Forest Service, 1995).

[13] Smith, D. M., Larson, B. C., Kelty, M. J. & Ashton, P. M. S. *The Practice of Silviculture: Applied Forest Ecology*. (Wiley and Sons, 1997).

[14] Janas PS, Brand DG (1988). Comparative growth and development of planted and natural stands of jack pine. Forest. Chron..

[15] Lo-Cho, C. N., Chung, H. H., Chiu, C. M., Chou, C. F. & Lo, S. S. Effects of thinning and pruning onTaiwania (Taiwania cryptomerioides Hayata) plantations. *Bull. Taiwan For. Res. Inst. New Series* **6**(2), 155–168 (1991).

[16] Raulier F, Pothier D, Bernier PY (2003). Predicting the effect of thinning on growth of dense balsam fir stands using a process-based tree growth model. Can. J. For. Res..

[17] Wang SY, Chang FC, Lee IC, Yang SL, Lin FC (2005). The study on the effective utilization of Japanese Cedar thinned logs. Jour. Exp. For. Nat. Taiwan Univ..

[18] Ohashi M, Gyokusen K, Saito A (1999). Measurement of carbon dioxide evolution from a Japanese cedar (Cryptomeria japonica D. Don) forest floor using an openflow chamber method. For. Ecol. Mgmt..

[19] Lee J, Morrison IK, Leblanc JD, Dumas MT, Cameron DA (2002). Carbon stock in trees and regrowth vegetation affected by clearcut and partial cut harvesting in a second-growth boreal mixed wood. For. Ecol. Mgmt..

[20] Laporte MF, Duchesne LC, Morrison IK (2003). Effect of clearcutting, selection cutting, shelterwood cutting and microsites on soil surface CO_2_ efflux in a tolerant hardwood ecosystem of northern Ontario. For. Ecol. Mgmt..

[21] Concilio A (2005). Soil respiration response to prescribed burning and thinning in mixed conifer and hardwood forests. Can. J. For. Res..

[22] Chen, L. C., Huang, G. M., Lin, J. S. & Chiou, C. R. Growing stock and growth estimation of Taiwania plantations in the Liukuei area. *Taiwan J. For. Sci*. **12**(3), 319–327 (1997).

[23] Lin, C. J., Chiu, C. M. & Wang, S. Y. Effects of thinning and pruning on wood density, form ratio, heartwood ratio, and sapwood width ofTaiwania (Taiwania cryptomerioides) plantation in Lukui area. *Quart. J. Chinese For*. **35**(1), 75–84 (2002).

[24] TFRI (Taiwan Forestry Research Institute). *Climate data of Liukuei station, TFRI* (TFRI Press, 1998).

[25] Dudley NS, Fownes JH (1992). Preliminary biomass equations for eight species of fastgrowing tropical trees. J. Trop. For. Sci..

[26] Lin, K. C., Wang, C. P., Huang, C. M., Horng, F. W. & Chiu, C. M. Estimates of biomass and carbon storage in two Taiwania plantations of the Lukui experimental forest. *Taiwan J. For. Sci*. **18**(2), 85–94 (2003).

[27] Brown S (2002). Measuring carbon in forests: current status and future challenges. Environ. Pollut..

[28] Lin, K. C., Huang, C. M., Wang, C. P. & Chang, N. H. Carbon and nitrogen accumulation and distribution in Taiwania plantations of the Liukuei Experimental Forest. *Taiwan J. For. Sci*. **19**(3), 225–235 (2004).

[29] Hou L, Li Z, Luo C, Bai L, Dong N (2016). Optimization forest thinning measures for carbon budget in a mixed pine-oak stand of the Qingling Mountains, China: A Case study. Forests.

[30] Schroeder P (1991). Can intensive management increase carbon storage in forests?. Environmental Mgmt..

[31] Alvarez S, Ortiz C, Díaz-Pinés E, Rubio A (2016). Influence of tree species composition, thinning intensity and climate change on carbon sequestration in Mediterranean mountain forests: A case study using the CO_2_ Fix model. Mitig. Adapt. Strat. Gl..

[32] Dewar RC, Cannell MGR (1992). Carbon stock in the trees, products and soils of forest plantations: an analysis using UK examples. Tree Physiol..

[33] Row, C. Effects of selected forest management options on carbon storage in *Forests and global change. Vol 2: forest management opportunities for mitigating carbon emissions* (Eds Sampson, N. & Hair, D.) 59–90 (American Forests, 1996).

[34] Strich, S. Carbon mitigation potential of German forestry considering competing forms of land use in *Carbon dioxide in forestry and wood industry* (Eds Kohlmaier, G.K., Weber, M., & Houghton, R.A.) 125–135 (Springer-Verlag, 1998).

[35] Vesterdal L, Dalsgaard M, Felby C, Raulund-Rasmussen K, Jorgensen BB (1995). Effects of thinning and soil properties on accumulation of carbon, nitrogen and phosphorus in the forest floor of Norway spruce stands. For. Ecol. Mgmt..

[36] Nilsen P, Strand LT (2008). Thinning intensity effects on carbon and nitrogen stores and fluxes in a Norway spruce (Picea abies (L.) Karst.) stand after 33 years. For. Ecol. Mgmt..

[37] Li S, Li S, Huang M (2017). Effects of thinning intensity on carbon stocks and changes in larch forests in China northeast forest region. Journal of Resources and Ecology.

[38] Ruiz-Peinado R, Bravo-Oviedo A, Montero G, del Río M (2016). Carbon stocks in a Scots pine afforestation under different thinning intensities management. Mitig. Adapt. Strat. Gl..

[39] Balboa-Murias MA, Rodríguez-Soalleiro R, Merino A, Álvarez-González JA (2006). Temporal variations and distribution of carbon stocks in aboveground biomass of radiata pine and maritime pine pure stands under different silvicultural alternatives. For. Ecol. Mgmt..

[40] Liu, S. H. *Forest management* (National Chung Hsing University Press, 1976).

